# Delivery of pDNA to the Lung by Lipopolyplexes Using *N*-Lauroylsarcosine and Effect on the Pulmonary Fibrosis

**DOI:** 10.3390/pharmaceutics13111983

**Published:** 2021-11-22

**Authors:** Tomoaki Kurosaki, Hiroki Kanda, Junya Hashizume, Kayoko Sato, Hitomi Harasawa, Tadahiro Nakamura, Hitoshi Sasaki, Yukinobu Kodama

**Affiliations:** 1 Department of Hospital Pharmacy, Nagasaki University Hospital, 1-7-1 Sakamoto, Nagasaki 852-8501, Japan; kurosaki@nagasaki-u.ac.jp (T.K.); hassy1984-ngs@umin.net (J.H.); satokayo@nagasaki-u.ac.jp (K.S.); harasawa-ngs@umin.ac.jp (H.H.); t-nakamr@nagasaki-u.ac.jp (T.N.); 2 Graduate School of Biomedical Sciences, Nagasaki University, 1-7-1 Sakamoto, Nagasaki 852-8588, Japan; bb30115010@ms.nagasaki-u.ac.jp; 3 Institute of Tropical Medicine, Nagasaki University, 1-12-4 Sakamoto, Nagasaki 852-8523, Japan; sasaki@nagasaki-u.ac.jp

**Keywords:** gene delivery, shRNA, nanoparticles, pulmonary fibrosis, *N*-lauroylsarcosine

## Abstract

In a previous study, we constructed a lung-targeting lipopolyplex containing polyethyleneimine (PEI), 1,2-di-*O*-octadecenyl-3-trimethylammonium propane (DOTMA), and *N*-lauroylsarcosine (LS). The lipopolyplex exhibited an extremely high gene expression in the lung after intravenous administration. Here, we optimized the lipopolyplex and used it to deliver a TGF-β1 shRNA to treat refractory pulmonary fibrosis. We constructed several lipopolyplexes with pDNA, various cationic polymers, cationic lipids, and LS to select the most effective formulation. Then, the pDNA encoding shRNA against mouse TGF-β1 was encapsulated in the lipopolyplex and injected into mice with bleomycin-induced pulmonary fibrosis. After optimizing the lipopolyplex, dendrigraft poly-L-lysine (DGL) and DOTMA were selected as the appropriate cationic polymer and lipid, respectively. The lipopolyplex was constructed with a pDNA, DGL, DOTMA, and LS charge ratio of 1:2:2:4 showed the highest gene expression. After intravenous administration of the lipopolyplex, the highest gene expression was observed in the lung. In the in vitro experiment, the lipopolyplex delivered pDNA into the cells via endocytosis. As a result, the lipopolyplex containing pDNA encoding TGF-β1 shRNA significantly decreased hydroxyproline in the pulmonary fibrosis model mice. We have successfully inhibited pulmonary fibrosis using a novel lung-targeting lipopolyplex.

## 1. Introduction

Gene therapy to the lungs has been studied in many genetic and refractory lung diseases, such as idiopathic pulmonary fibrosis (IPF) [1], asthma [2], and several types of lung cancer [3,4].

IPF is also a progressive, fatal lung disease. Nintedanib and pirfenidone, approved by the Food and Drug Administration to treat IPF in 2014 based on a positive phase 3 trial [5], are recommended in the 2015 ATS/ERS/JRS/ALAT clinical practice guidelines. These drugs are used to suppress IPF progression; however, they are not radical treatments. Furthermore, they cause side effects, such as critical nausea and diarrhea [6,7], in many patients. Therefore, gene therapy is increasingly considered as a new treatment; because it works via a different mechanism from that of existing drugs, it can be a radical treatment.

The transforming growth factor-β (TGF-β) family and platelet-derived growth factor are factors for exacerbation in IPF [8,9]. For example, TGF-β1 is important for promoting fibroblast activation, migration, infiltration, or hyperplasia. In addition, the excess extracellular matrix produced by TGF-β1 activation significantly contributes to IPF progression [10,11,12]. Moreover, it was reported in a review that suppressing fibrosis-related genes such as TGF-β1 with small interfering RNA (siRNA) or short hairpin RNA (shRNA) administered in the respiratory route inhibits IPF in model animals [13].

However, therapeutic delivery via the respiratory route to IPF patients is challenging due to the decreased respiratory function, thickened epithelial cells in the airway and lung, and increased mucosal secretion [14,15]. Therefore, there is a need for a delivery strategy that delivers gene medicine to the lung selectively after systemic administration.

In a previous study, we found that the gene delivered by a lipopolyplex containing *N*-lauroylsarcosine (LS) had a high level of expression in the lung after intravenous administration [16]. LS was reported as a low-toxicity biodegradable surfactant [17]. This lipopolyplex comprised pDNA, polyethylenimine (PEI), 1,2-di-*O*-octadecenyl-3-trimethylammonium propane (DOTMA), and LS. Different components presumably affect the contribution rates to gene expression in the lung. Multivariate analysis revealed that LS had the highest contribution rate to gene expression in the lung. Still, the biocompatibility and gene expression efficiency of the lipopolyplex should be improved before clinical application.

Thus, in this study, we constructed lipopolyplexes with various cationic polymers and lipids. Then, we compared their gene expressions before selecting an appropriate lipopolyplex. Next, we tested the lipopolyplex containing pDNA encoding TGF-β1 shRNA (psh-TGF-β1) as a treatment in the mouse model of IPF.

## 2. Materials and Methods

### 2.1. Chemicals

PEI (branched form, average molecular weight (MW): 25,000 Da) and LS (Aldrich Chemical Co., Milwaukee, WI, USA); dendrigraft poly-l-lysine (DGL) (Generation 5, MW: 172,300 Da, 963 lysine groups) (COLCOM SAS, Montpellier, France); DOTMA (Avanti Polar Lipid, Inc., Alabaster, AL, USA); 1,2-dioleoyl-3-trimethylammonium propane (DOTAP) and 1,2-dioleoyl-3-dimethylammonium-propane (DODAP) (NOF America Corporation, White Plains, NY, USA); and poly-l-lysine (PLL), poly-l-arginine (PLA), and cholesteryl 3β-*N*-(dimethylaminoethyl)carbamate hydrochloride (DC-chol) (Sigma-Aldrich, St. Louis, MO, USA) were purchased. In addition, fetal bovine serum (FBS) (Biological Industries Ltd., Kibbutz Beit Haemek, Israel); the 100× antibiotic solution containing penicillin G, streptomycin, and l-glutamine (Wako Pure Chemical Industries, Ltd., Osaka, Japan); and Dulbecco’s Modified Eagle Medium (DMEM) (Nissui Pharmaceutical Co., Ltd., Tokyo, Japan) were also obtained.

### 2.2. Construction of pDNA

pCMV-Luc was constructed by subcloning the HindIII/XbaI firefly luciferase cDNA fragment from pGL3-control (Promega, Madison, WI, USA) into the polylinker of pcDNA3 (Invitrogen, Carlsbad, CA, USA). The pDNA vectors encoding negative control shRNA (psh-NTC, Cat No: TR30013) and TGF-β1 shRNA (psh-TGF-β1, Cat No: TG502269; OriGene Technologies, Inc., Rockville, MD, USA) were purchased. Cy3-pDNA was prepared using the Label IT Cy3 Labeling Kit (Takara Bio Inc., Shiga, Japan).

### 2.3. Preparation of the Lipopolyplexes

pDNA solution (1 mg/mL) and various cationic polymer solutions (10 mg/mL) were prepared in 5% glucose solution. Cationic lipids and LS were dissolved in ethanol as 10 mg/mL. The theoretical charge ratio of the cationic polymers to pDNA complexes was calculated as the molar ratio of cationic polymer nitrogen to pDNA phosphate. An appropriate amount of a stock cationic polymer solution was thoroughly mixed with a diluted solution of 1 mg/mL pDNA to prepare polyplexes (complexes of pDNA and cationic polymer) at a charge ratio of 0.5, 1, 2, or 4. The solutions were incubated at room temperature for 15 min. When preparing the lipopolyplexes, the theoretical charge ratio of cationic lipids to pDNA was calculated as the molar ratio of cationic lipid nitrogen to pDNA phosphate. Meanwhile, the theoretical charge ratio of LS to pDNA was calculated as the molar ratio of LS carboxylate to pDNA phosphate.

After the addition of cationic lipids in ethanol to polyplexes at a charge ratio of 0.5, 1, 2, or 4, the mixtures were incubated at room temperature for 15 min. In addition, the LS ethanol solution was added to the lipopolyplex at a charge ratio of 1, 2, 4, or 6, and the mixtures were incubated at room temperature for 15 min. Then, the mixtures contained approximately 10–24% (*v*/*v*) of ethanol.

For fluorescence microscopy, a lipopolyplex was constructed with Cy3-pDNA as described above. Finally, NBD-PE was added to the lipopolyplex to stain its lipid layer.

### 2.4. Animals

All animal care and experimental procedures were performed according to the Guidelines for Animal Experimentation of Nagasaki University with approval from the Institutional Animal Care and Use Committee (1710191419, 19 October 2017). All animals were housed under a 12 h dark–light cycle with ad libitum food and water. Five-week-old male ddY mice (Japan SLC, Inc., Shizuoka, Japan) were acclimatized to their new environment for at least 1 day before the experiments. Three to five mice were used for each group.

### 2.5. Physicochemical Characteristics of Lipopolyplexes

Lipopolyplexes were diluted with water and the particle sizes, polydispersity indexes (PDI), and ζ-potentials of various lipopolyplexes were measured using a Zetasizer Nano ZS (Malvern Instruments, Ltd., Malvern, UK). The particle sizes were shown as the ζ-average particle sizes.

### 2.6. In Vivo Gene Expression Experiments

Lipopolyplexes containing 40 μg of pCMV-Luc were injected into the tail vein of the mice at 200 μL per mouse. Then, the mice were sacrificed 6 h after administration. Next, their livers, kidneys, spleens, hearts, and lungs were dissected and homogenized with a lysis buffer (pH 7.8 and 0.1 M Tris/HCl buffer containing 0.05% Triton X-100 and 2 mM ethylenediaminetetraacetic acid) using a homogenizer (Omni TH-115, Yamato Scientific Co., Ltd., Tokyo, Japan). After the homogenates were centrifuged at 20,600× g for 5 min, the supernatants were used for luciferase assay.

### 2.7. Luciferase Assay

A lysate sample (10 µL) was mixed with 50 μL of luciferase assay buffer (Picagene, Toyo Ink, Tokyo, Japan) and immediately measured using a luminometer (Lumat LB 9507, EG&G Berthold, Bad Wildbad, Germany). The luciferase activity was indicated in relative light units (RLU) per g tissue for the in vivo experiments. The luciferase activities in the liver, kidney, spleen, heart, and lung dissected from the untreated mice were below 10^5^ RLU/g tissue.

### 2.8. In Vitro Cellular Uptake of pDNA/DGL/DOTMA/LS

Lewis lung carcinoma LLC cells (Cell Resource Center for Biomedical Research, Tohoku University, Sendai, Japan) were maintained in DMEM containing 10% FBS in a humidified atmosphere with 5% CO_2_ at 37 °C before being plated onto four-well plates (Corning, Inc., Corning, NY, USA) at 3.0 × 10⁴ cells/well and in 500 µL medium. Then, the cells were treated with lipopolyplex containing Cy3-pDNA and NBD-PE after 24 h of preincubation to visualize the uptake of pDNA/DGL/DOTMA/LS. After 2 h of incubation, a culture medium containing 5 µg/mL Hoechst 33342 was added to the cells, and the cells were incubated at 37 °C in the dark for 15 min. The cells and fluorescence of Cy3-pDNA, NBD-PE, and Hoechst 33342 were measured by a BZ-X Analyzer (KEYENCE, Osaka, Japan) using a fixed-magnification lens (20×).

### 2.9. In Vivo Hydroxyproline Quantification

We established an IPF model in mice by intrabronchially administering 5 mg/kg bleomycin.

The mice were intravenously administered 5% glucose solution with pDNA/DGL/DOTMA/LS containing 40 μg of psh-TGF-β1 or psh-NTC 6 h before treatment with bleomycin. One week later, these mice were again treated with the same lipopolyplexes. Two weeks after the administration of bleomycin, the lungs were removed from the mice to measure the hydroxyproline levels using a Hydroxyproline Assay Kit (Sigma-Aldrich, St. Louis, MO, USA).

### 2.10. Statistical Analysis

Multiple comparisons among the groups were performed using the Fisher’s LSD test. A difference with a *p*-value less than 0.05 was considered statistically significant.

## 3. Results

### 3.1. Effect of Cationic Polymers on Particle Size and ζ-Potential of Lipopolyplexes

We used four kinds of cationic polymers, namely PEI, PLL, PLA, and DGL. In addition, the pDNA, a cationic polymer, DOTMA, and LS were mixed at a charge ratio of 1:2:2:4. The particle sizes of all the lipopolyplexes were around 200 nm, and the ζ-potential was around 15 mV in all cases (Table 1).

### 3.2. Effect of Cationic Polymers on the Lung Transfection Efficiency of Lipopolyplexes

**Figure 1:**
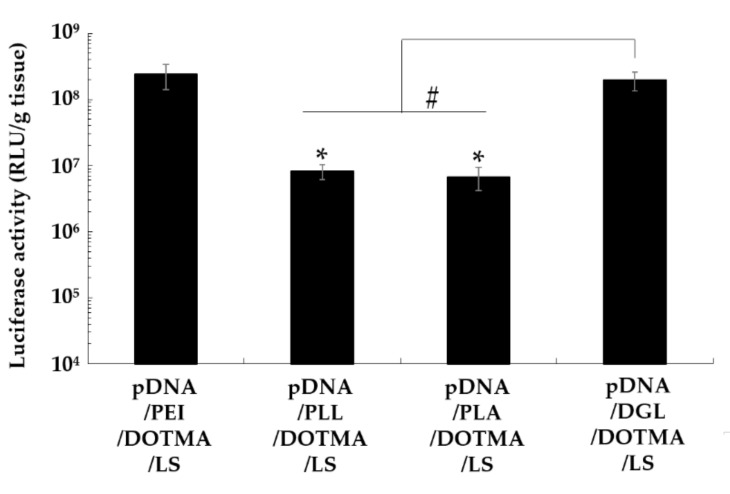
Effect of cationic polymers on the luciferase gene expression in the lung after the intravenous administration of various lipopolyplexes. Each value is expressed as mean ± S.E. (*n* = 4). * *p* < 0.05, comparison with pDNA/PEI/DOTMA/LS; # *p* < 0.05.

The lipopolyplexes containing PEI, PLL, PLA, or DGL were administered to the mice to compare their gene expression in the lung. pDNA/PEI/DOTMA/LS exhibited a high luciferase activity of more than 1 × 10^8^ RLU/g tissue (Figure 1). Contrarily, the lipopolyplexes containing PLL and PLA exhibited significantly lower luciferase activity than pDNA/PEI/DOTMA/LS (*p* < 0.05). pDNA/DGL/DOTMA/LS also showed a luciferase gene expression comparable to that of pDNA/PEI/DOTMA/LS. DGL is known to be a biodegradable cationic polymer and more suitable for in vivo applications than PEI. Therefore, we used DGL as a component of the lipopolyplex in the subsequent experiments.

### 3.3. Effect of Cationic Lipids on Particle Size and ζ-Potential of Lipopolyplexes

The sizes of the lipopolyplexes containing DOTMA, DOTAP, or DC-chol were approximately 210 nm. Contrarily, pDNA/DGL/DODAP/LS was aggregated. In addition, the ζ-potentials of the lipopolyplexes containing DOTMA and DOTAP were around 12 mV. Moreover, the ζ-potentials of the lipopolyplexes containing DODAP and DC-chol were about 3 and 29 mV, respectively (Table 2).

### 3.4. Effect of Cationic Lipids on the Lung Transfection Efficiency of Lipopolyplexes

**Figure 2:**
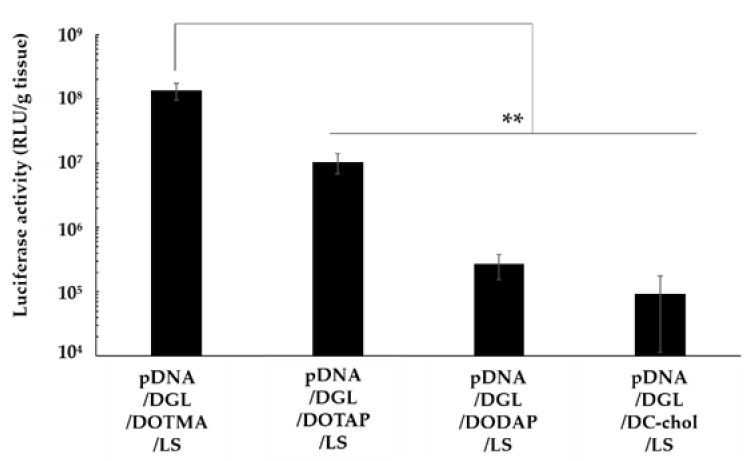
Effect of cationic lipids on the luciferase gene expression in the lung after the intravenous administration of various lipopolyplexes. Each value is expressed as mean ± S.E. (*n* = 4). ** *p* < 0.01.

The lipopolyplexes containing DOTMA, DOTAP, DODAP, or DC-chol were intravenously administered to the mice, and their respective gene expression in the lung was determined. pDNA/DGL/DOTMA/LS exhibited a significantly higher luciferase activity compared with other lipopolyplexes (*p* < 0.01) (Figure 2). Therefore, we used DOTMA as a component of the lipopolyplex in the subsequent experiments.

### 3.5. Effect of Charge Ratio on the Gene Expression of pDNA/DGL/DOTMA/LS in the Lung

**Figure 3:**
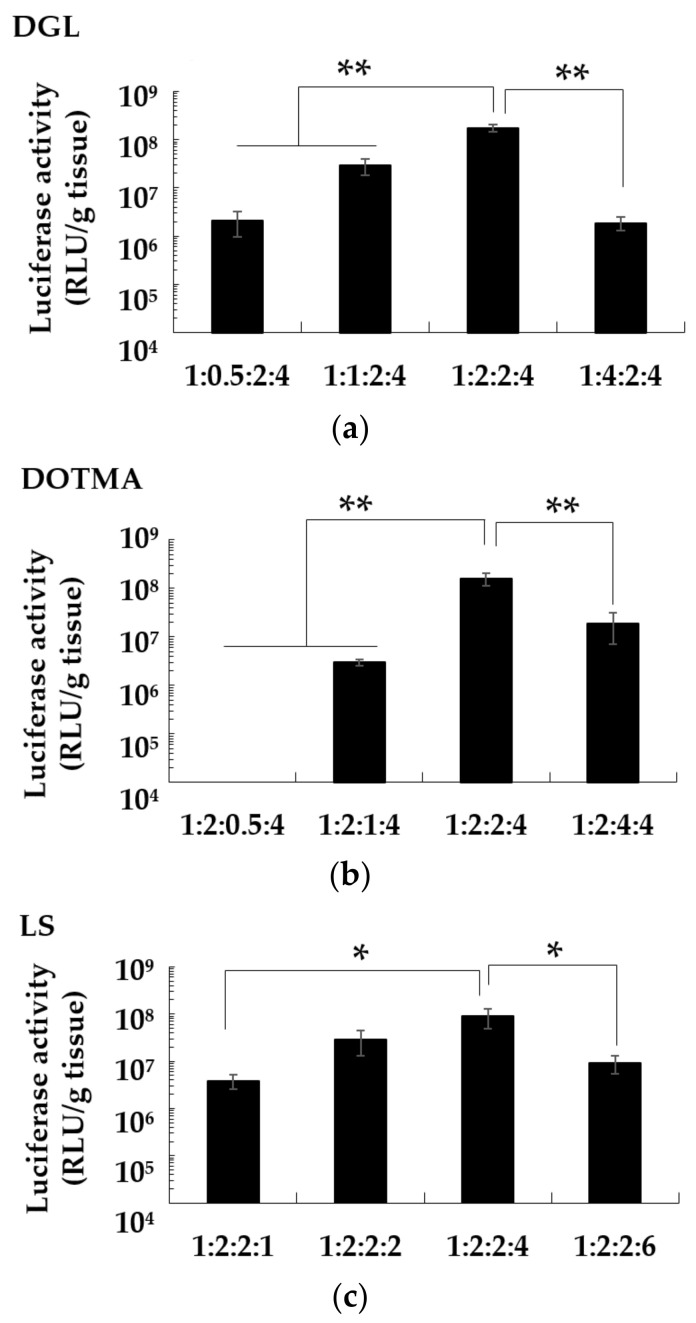
Effect of charge ratios of DGL (**a**), DOTMA (**b**), or LS (**c**) on the luciferase gene expression in the lung after the intravenous administration of the pDNA/DGL/DOTMA/LS. Each value is expressed as mean ± S.E. (*n* = 3–4). * *p* < 0.05; ** *p* < 0.01.

pDNA/DGL/DOTMA/LS lipopolyplexes with different charge ratios were constructed, and their transfection efficiency in the lung was determined. The highest luciferase activity was observed at the charge ratio of 1:2:2:4 (in Figure 3); therefore, the pDNA/DGL/DOTMA/LS lipopolyplex with this charge ratio was used in the subsequent experiments.

### 3.6. In Vivo Distribution of Gene Expression by pDNA/DGL/DOTMA/LS

**Figure 4:**
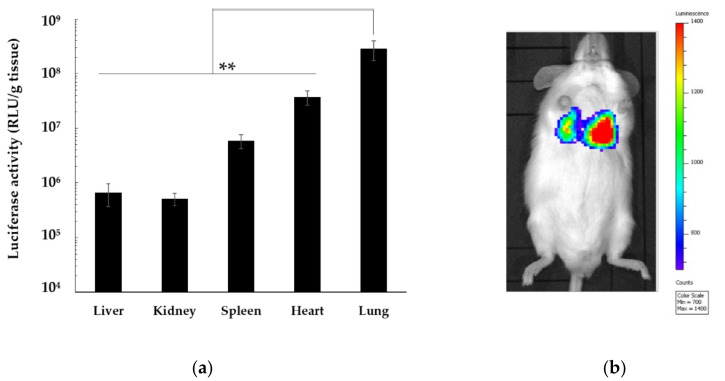
In vivo distribution of gene expression by pDNA/DGL/DOTMA/LS. pDNA/DGL/DOTMA/LS was intravenously administered to the mice. Six hours after the administration, in vivo bioluminescence images were taken, and five organs were removed. (**a**) The luciferase activity in the organs was measured. (**b**) Bioluminescence imaging of a mouse was performed using an in vivo imaging system. Each value is expressed as mean ± S.E. (*n* = 4). ** *p* < 0.01.

The gene expression in the liver, kidney, spleen, heart, and lung was determined after the intravenous administration of pDNA/DGL/DOTMA/LS. The luciferase activity in the lungs was significantly higher than that in the liver, kidney, spleen, and heart (*p* < 0.01) (Figure 4a). In addition, according to in vivo bioluminescence image, the highest luminescence was observed around the lung (Figure 4b).

### 3.7. Intracellular Uptake Pathway of pDNA/DGL/DOTMA/LS

**Figure 5:**
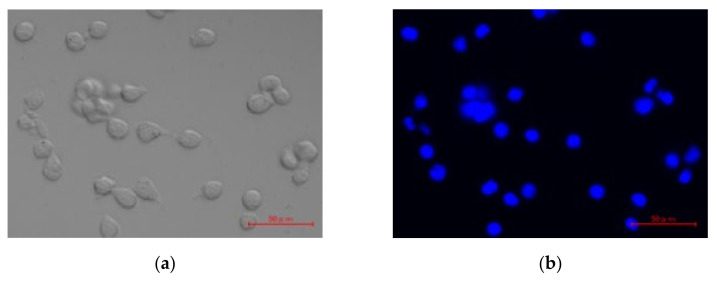

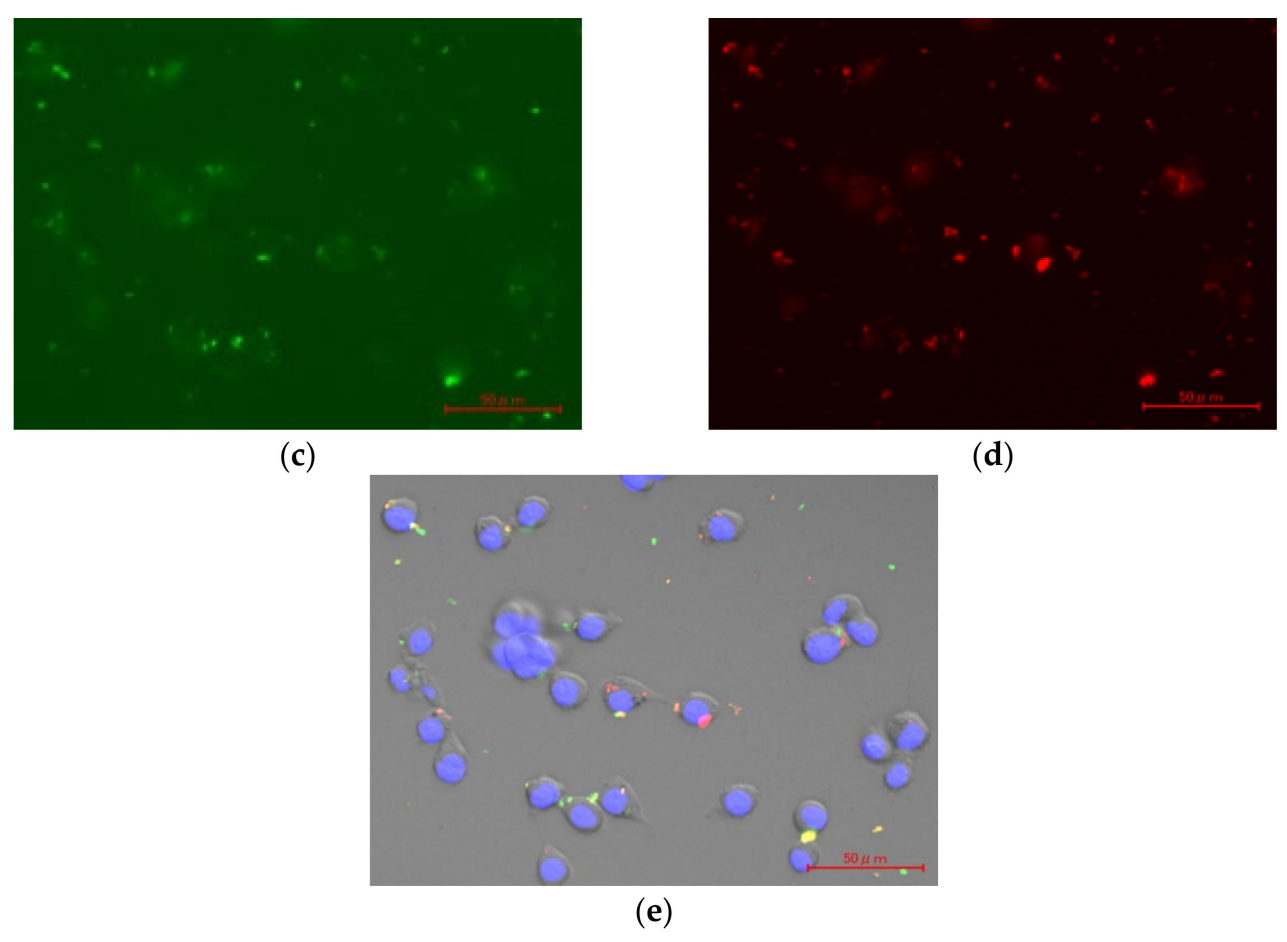
Intracellular distribution of pDNA/DGL/DOTMA/LS. A phase-contrast image (**a**), nuclei staining with Hoechst 33342 (**b**), lipid layer stained with NBD-PE (**c**), Cy3-pDNA (**d**), and merged image (**e**) were taken at 20× magnification. Red scale bar, 50 µm.

The intracellular uptake of the lipopolyplexes in LLC cells was observed using a fluorescence microscope (Figure 5). The nucleus was stained with Hoechst 33342 (blue), the lipid layer of pDNA/DGL/DOTMA/LS was stained with NBD-PE (green), and pDNA was labeled with Cy3 (red). Many green and red dots were observed in the cytosol, and yellow dots, indicating the complexes of the green-labeled lipid layer and red-labeled pDNA, were detected in the merged images (Figure 5e).

### 3.8. Effect of pDNA/DGL/DOTMA/LS Containing psh-TGF-β1 on the Mice with IPF

**Figure 6:**
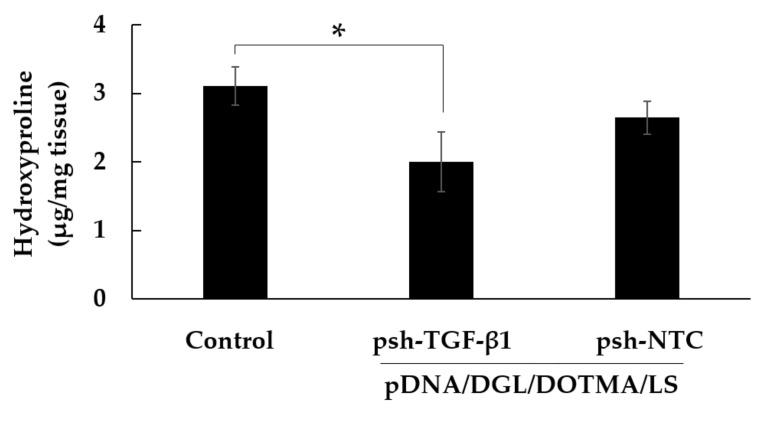
Effect of pDNA/DGL/DOTMA/LS containing psh-TGF-β1 on the amount of hydroxyproline in the lung of the mice with bleomycin-induced IPF. Each value is expressed as mean ± S.E. (*n* = 4–5); * *p* < 0.05.

The mice with bleomycin-induced IPF were generated and treated with pDNA/DGL/DOTMA/LS containing psh-TGF-β1. The level of fibrosis was determined by measuring the hydroxyproline in the lung. The amount of hydroxyproline was significantly reduced in the group receiving the pDNA/DGL/DOTMA/LS containing psh-TGF-β1 as compared with the control group receiving 5% glucose solution (*p* < 0.05, Figure 6). Meanwhile, the pDNA/DGL/DOTMA/LS containing psh-NTC had a negligible effect on the mice with induced IPF.

## 4. Discussion

The lung is an essential organ as it exchanges carbon dioxide for oxygen and adjusts the blood pH. There are several intractable lung diseases, such as IPF and pulmonary arterial hypertension. Various drugs have been developed for those lung diseases; however, none of them is a radical treatment. The suppression of TGF-β1 in the lung using siRNA or shRNA was reported to be one promising treatment for IPF in a preclinical study [9]. Although the respiratory administration of siRNA and shRNA is an effective and safe delivery method, it can be physically difficult for patients with decreased lung function [14]. Therefore, there is a need for a different method of delivering siRNA and shRNA to the lung.

In a previous study, we found that a pDNA/PEI/DOTMA/LS lipopolyplex exhibited an extremely high gene expression in the lung at 6 h after administration, and LS contributed to the lung gene expression as 76.7% of the contribution index [16]. The contribution indexes of PEI and DOTMA were low; however, different kinds of cationic polymers and lipids might affect the transfection efficiency of the lipopolyplex. In this study, we constructed various lipopolyplexes with different cationic polymers and lipids and compared their transfection efficiencies in the lung.

To examine the effect of a cationic polymer on the transfection efficiency of a lipopolyplex, we constructed lipopolyplexes with PEI, PLL, PLA, or DGL. Biodegradable PLL, PLA, and DGL have been widely used for nonviral gene delivery. After intravenous administration, the lipopolyplexes containing PLL and PLA demonstrated significantly lower lung gene expressions compared with pDNA/PEI/DOTMA/LS. Meanwhile, pDNA/DGL/DOTMA/LS showed a level of gene expression in the lung comparable to that of pDNA/PEI/DOTMA/LS. However, there was a slight difference in the physicochemical properties among those lipopolyplexes.

In the in vitro experiments, pDNA/DGL/DOTMA/LS was shown to be taken by LLC cells via endocytosis, not membrane fusion, as the lipopolyplex was absorbed by the cells as a complex of pDNA and lipid. PEI destabilizes endosomal membranes with its pH buffering capacity, thus improving the cytoplasmic transfer of pDNA [18,19]. DGL also has a high pH buffering capacity because the spatial density of cations is increased by the sterically crowded structure [20]. Contrarily, PLL and PLA demonstrate low endosome escape because of their low pH buffering capacities [21]. These reports suggest that the pH buffering capacity of a cationic polymer is important for the transfection efficiencies of the lipopolyplexes.

We constructed four kinds of lipopolyplexes containing DOTMA, DOTAP, DODAP, and DC-chol. pDNA/DGL/DODAP/LS was aggregated and PDI of the lipopolyplex was over 0.7; however, other lipopolyplexes were stably constructed. Particles with a strong charge repel each other. Meanwhile, particles with a nearly neutral charge have a weak repulsive force, and the bonding between these particles is considered to be induced by hydrophobic interactions. The cationic charge of DODAP due to the secondary amine is insufficient for constructing stable nanoparticles at pH 7.4. The lipopolyplexes containing DOTMA or DOTAP had lower ζ-potentials than pDNA/DGL/DC-chol/LS, likely due to the formation of lipid bilayers of the cationic lipids. After intravenous administration, pDNA/DGL/DODAP/LS exhibited low gene expressions in the lung. The low gene expression of pDNA/DGL/DODAP/LS was likely caused by aggregation. Meanwhile, lipopolyplexes containing DOTMA or DOTAP showed high gene expressions, and pDNA/DGL/DOTMA/LS showed the highest gene expression. The uptake mechanism of pDNA/DGL/DOTMA/LS was demonstrated to be endocytosis, not membrane fusion. DOTMA and DOTAP destabilize membranes by acidulation after endocytosis [22,23,24]. Contrarily, pDNA/DGL/DC-chol/LS was found to have low gene expression despite its strong ζ-potential. The minimal membrane destabilizing effect of DC-chol [25] likely caused the low gene expression of pDNA/DGL/DC-chol/LS. Cationic lipids with high membrane fusion efficiency could show different results, and we would like to determine the effect of membrane fusion lipids on the transfection efficiency of the lipopolyplexes in the future. The difference in gene expression between the lipopolyplexes containing DOTMA and DOTAP might be due to their different chemical structures. DOTMA has ether bonds, whereas DOTAP has ester bonds. Ester bonds are susceptible to hydrolysis inside the body [26]. Thus, the stability of DOTMA might contribute to the high transfection efficiency of pDNA/DGL/DOTMA/LS in the lung.

Then, the optimal charge ratio for the components of pDNA/DGL/DOTMA/LS was determined. In a previous study, we demonstrated that the pDNA/PEI/DOTMA/LS ratio of 1:2:2:4 was the optimal charge ratio for pDNA/PEI/DOTMA/LS [16]. Next, we found that the charge ratio of 1:2:2:4 for pDNA/DGL/DOTMA/LS was also optimal. After optimizing the lipopolyplexes, we determined the detailed in vivo transfection efficiency of pDNA/DGL/DOTMA/LS. After intravenous administration of pDNA/DGL/DOTMA/LS, the gene expression in the lung was significantly higher than that in other organs, such as the liver, kidney, spleen, and heart. The highest gene expression in the lung was also observed using an in vivo imaging system.

Next, we tested a novel therapeutic method by applying pDNA/DGL/DOTMA/LS containing pDNA encoding shRNA against TGF-β1 (psh-TGF-β1) to mice with induced IPF. shRNA sequences are usually encoded in a pDNA vector, and shRNA is transcribed from the pDNA into the transfected cells. After transcription, shRNA is incorporated into the RNA-induced silencing complex and cleaves targeted mRNA for a long period [27,28,29]. Therefore, pDNA/DGL/DOTMA/LS containing psh-TGF-β1 could decrease the TGF-β1 protein expression in the lungs of mice with induced IPF. Here, we successfully decreased the amount of hydroxyproline in the lung via intravenous administration of the pDNA/DGL/DOTMA/LS containing psh-TGF-β1. The decrease in collagen accumulation suggests that fibrosis can be inhibited in the lung. In addition, pDNA/DGL/DOTMA/LS containing psh-NTC showed little effect on the hydroxyproline levels in the lung, indicating that the components of the lipopolyplex did not affect IPF. In the previous studies, nintedanib and pirfenidone were reported to decrease the lung hydroxyproline level in the IPF model mice by approximately two-thirds in certain conditions [30,31]. In this experiment, pDNA/DGL/DOTMA/LS containing psh-TGF-β1 showed an effect comparable to that of those medicines. These results indicate that pDNA/DGL/DOTMA/LS containing psh-TGF-β1 is suitable for treating IPF. However, further studies should be conducted to investigate the mechanisms and side effects of pDNA/DGL/DOTMA/LS containing psh-TGF-β1 in detail.

## Figures and Tables

**Table 1:** Effect of cationic polymers on the particle size and ζ-potential of the lipopolyplexes.

Complex	Size (nm)	PDI	ζ-Potential (mV)
pDNA/PEI/DOTMA/LS	185.1 ± 5.87	0.24 ± 0.01	16.7 ± 0.50
pDNA/PLL/DOTMA/LS	169.7 ± 4.71	0.23 ± 0.01	14.5 ± 0.10
pDNA/PLA/DOTMA/LS	179.5 ± 2.50	0.21 ± 0.01	17.8 ± 0.46
pDNA/DGL/DOTMA/LS	211.6 ± 2.20	0.26 ± 0.01	11.3 ± 0.36

Data are expressed as mean ± S.D.

**Table 2:** Effect of cationic lipids on particle size and ζ-potential of lipopolyplexes.

Complex	Size (nm)	PDI	ζ-Potential (mV)
pDNA/DGL/DOTMA/LS	211.6 ± 2.20	0.25 ± 0.02	11.3 ± 0.36
pDNA/DGL/DOTAP/LS	221.5 ± 10.97	0.25 ± 0.03	12.3 ± 0.70
pDNA/DGL/DODAP/LS	Aggregated	0.74 ± 0.04	2.93 ± 0.70
pDNA/DGL/DC-chol/LS	202.7 ± 10.17	0.33 ± 0.03	28.9 ± 0.42

Data are expressed as mean ± S.D.

## Data Availability

Not applicable.
